# A New Look at the Spin Glass Problem from a Deep Learning Perspective

**DOI:** 10.3390/e24050697

**Published:** 2022-05-14

**Authors:** Petr Andriushchenko, Dmitrii Kapitan, Vitalii Kapitan

**Affiliations:** 1 National Center for Cognitive Research, ITMO University, bldg. A, Kronverksky Pr. 49, 197101 Saint Petersburg, Russia; madgfess@gmail.com; 2 Department of Theoretical Physics and Smart Technologies, Far Eastern Federal University, Russky Island, 10 Ajax Bay, 690922 Vladivostok, Russia; kapitan.vyu@dvfu.ru; 3 Institute of Applied Mathematics, Far Eastern Branch, Russian Academy of Science, 7 Radio, 690041 Vladivostok, Russia

**Keywords:** spin glass, Ising model, machine learning, deep neural network

## Abstract

Spin glass is the simplest disordered system that preserves the full range of complex collective behavior of interacting frustrating elements. In the paper, we propose a novel approach for calculating the values of thermodynamic averages of the frustrated spin glass model using custom deep neural networks. The spin glass system was considered as a specific weighted graph whose spatial distribution of the edges values determines the fundamental characteristics of the system. Special neural network architectures that mimic the structure of spin lattices have been proposed, which has increased the speed of learning and the accuracy of the predictions compared to the basic solution of fully connected neural networks. At the same time, the use of trained neural networks can reduce simulation time by orders of magnitude compared to other classical methods. The validity of the results is confirmed by comparison with numerical simulation with the replica-exchange Monte Carlo method.

## 1. Introduction

Spin glasses fundamentally differ from other lattice models by the presence of frustrations—strong competition of magnetic interactions, and disorder—freezing of atoms upon cooling. Due to these key features, spin glasses have long relaxation times, a rough energy landscape, and macroscopic degeneracy of ground states. This leads to the fact that the numerical simulation and even more so the analytical description of such systems becomes a challenging task. The first attempts at theoretical description of the spin glass model [1,2] encountered several difficulties. One of the difficulties was the lack of translational invariance in the spin glass systems. Another problem was the non-ergodicity of the spin glass phases due to the presence of many local energy minima, which are separated by high potential barriers. Hence, the problem of configurational averaging and the calculation of residual entropy follows. The problem is that the entropy of the system being in the true thermodynamic equilibrium should be equal to zero. However, this is not applicable to spin glasses, since the energy of the system may depend not only on the temperature, but also on the history of states of the sample. Thus, at the lowest temperatures, the so-called residual entropy can be observed [3]. It can be calculated through the degeneracy of the ground states. This parameter is one of the key parameters of systems with competing interactions.

Many processes that occur in spin glasses cannot be described within the framework of the classical theory of phase transitions and require new approaches. We would also like to note the relevance of such a problem since the approaches being developed to describe spin glasses lead to entirely new results far beyond theoretical physics. Such results include, for example, contributions to solving multiparameter optimization problems or problems in the area of associative and distributed memory [4,5,6].

Since the class of spin models on lattices described by Ising-like Hamiltonians can be solved analytically only in extremely rare cases [7], numerical probabilistic methods, such as different variations of the Monte Carlo method, are now most commonly used to describe the physics of spin glass [8,9,10,11]. However, properties of spin glasses, such as long relaxation time, rough energy landscape, macroscopic degeneracy of ground states, and the effects of critical slowing down, significantly decrease the efficiency of the Monte Carlo algorithms. The motion of the system in phase space is very slow, so it requires generation of an extremely large number of states to move to an equilibrium state. On the other hand, there has been exponential growth in computational power and the rapid development of Monte Carlo methods, leading to the use of these methods in almost all fields of physics [12,13,14,15]. It allows to partially offset the increasing complexity of calculations with the increasing size of the spin glass systems [16,17,18].

Simultaneously with the development of numerical Monte Carlo methods, the exponential growth of computational power led to the second revolution in the area of neural networks and the emergence of completely new architectures and approaches to neural network training—convolutional neural networks, autoencoders, constrained Boltzmann machines, etc. [19]. All of these approaches have dramatically reduced training time and increased the dimensionality of the tasks to be solved. This revolution has led to an unprecedented expansion of machine learning methods into all areas of life, business and science. In particular, machine learning methods have begun to be applied to statistical physics [20,21,22].

Several approaches have been proposed to study magnetic systems using machine learning. The first one is to use supervised machine learning algorithms to solve the problem of classifying the states of magnetic systems (with nearest-neighbors interaction on a square lattice) into thermodynamic phases. The input of such a model is the configuration of the magnetic system (states of all spins) obtained by Monte Carlo simulation, and the output of the model predicts the most probable phase where the configuration could appear [23,24,25]. The advantage of this approach is the ability to use convolutional neural networks, which are commonly used for image recognition tasks. It can be explained by the fact that such architectures naturally convey the fundamental properties of a square lattice—the spatial arrangement of spins and the interactions with the nearest four neighbors.

The second approach is to use supervised machine learning algorithms to solve the regression problem of searching for the lowest energy configurations (ground states) of spin systems with complex interactions, such as spin glass or spin ice. The problem of finding the lowest energy states of spin glasses is a key problem since such states are highly degenerate, often asymmetric, and separated by high energy barriers. However, such states have the highest probability at a lower temperature. Consequently, they are the ones that contribute the most to the statistical sum and thus to all the thermodynamic averages [26,27].

In this paper, we propose to solve a more general problem: using machine learning methods to solve the problem of regression of the basic thermodynamic characteristics 〈E〉,〈M〉 (average energy and magnetization) or any other system characteristics, as a function of temperature *T* for spin glasses on a square lattice. Widely known, there is Cybenko’s theorem on the universal approximator, proving that any continuous function of many variables can be approximated with a given accuracy by a feedforward neural network with one hidden layer [28]. For this purpose, we consider the spin glass as a weighted graph, in which the architecture of the graph corresponds to the lattice and the values of the edges correspond to the values of the exchange interaction. Thus, using a neural network, we are looking for a functional dependence between the spatial distribution of the exchange integral on the square lattice of the spin glass $J_{k}=f_{J}\left(x_{k},y_{k}\right)$ and the main average thermodynamic characteristics of the system 〈E〉,〈M〉. Here, $f_{J}$ is the function of the spatial distribution of spin glass bonds values, $J_{k}$ is the bond value, $x_{k},y_{k}$ is the bond coordinates for bond *k*. To solve this class of problems, it is relevant to find an architecture of a neural network that will be able to approximate this function most effectively using the spatial regularities of the lattice.

## 2. Model and Data

### 2.1. Spin Glass Model

**Figure 1:**
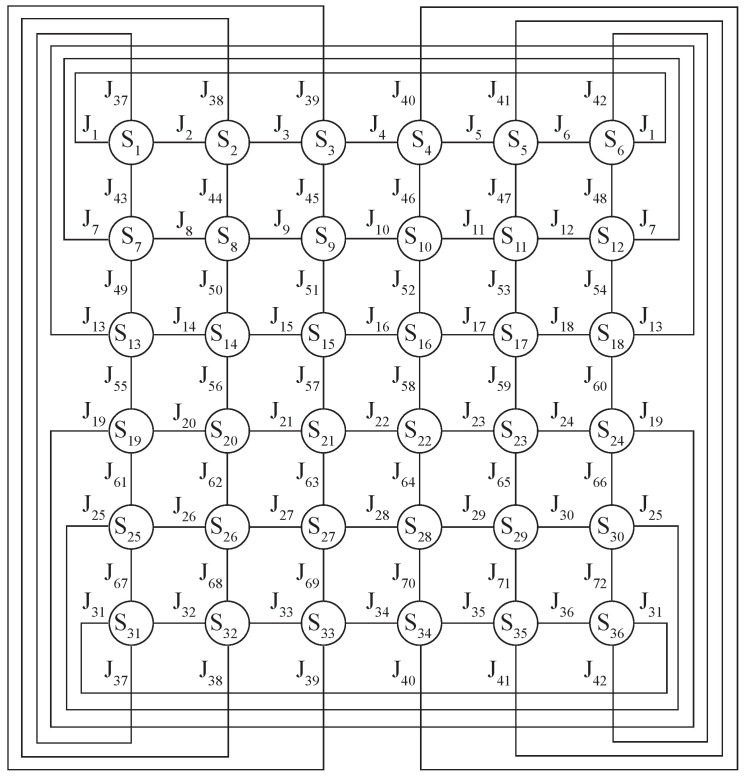
Example of the spin glass model on a square lattice 6×6 of Ising spins with a periodical boundaries condition. $S_{i}=\pm 1$—spins of the lattice, $J_{k}=\pm 1$—exchange integral.

In this paper, we consider spin glass models with periodic boundary conditions on a square lattice N=L×L, in which each spin is an Ising spin, i.e., it has two states $S_{i}=\pm 1$, and has four nearest neighbors, the interactions with which are determined by the exchange integral $J_{k}=\pm 1$ (see Figure 1). The standard Hamiltonian of such a system has the form:

(1) $$\begin{array}{c} H=-\sum_{<ij>}J_{k}S_{i}S_{j}, \end{array}$$

where $S_{i},S_{j}$ are the interacting spins that are nearest neighbors on the square lattice, <i,j> is the summation that occurs only by nearest neighbors, $J_{k}$ is the value of the exchange interaction between spins $S_{i}$ and $S_{j}$, the index k=k(i,j) is a function of i,j.

Mean energy 〈E〉 of the spin glass at temperature *T* is calculated by (2), and the mean magnetization 〈M〉 by (3):

(2) $$\begin{array}{c} {\langle E\rangle }_{T}=\frac{1}{N}{\langle H\rangle }_{T}, \end{array}$$

(3) $$\begin{array}{c} {\langle M\rangle }_{T}=\frac{1}{N}{\langle \sum_{i}S_{i}\rangle }_{T}. \end{array}$$

The spatial distribution of the exchange integral $J_{k}=f_{J}\left(x_{k},y_{k}\right)$ determines all macroscopic characteristics of spin glass. Such a description of the spin glass model, at first glance, may seem extremely simple. Since it is a classical spin model, it seems that numerical simulations can be performed quite easily with classical numerical Monte Carlo methods [29]. However, this is a misleading simplicity. There are many examples of extremely simple systems that present unpredictable behavior. Examples of such systems are given in Stephen Wolfram’s book [30], in which he described remarkably simple models that gave complex, non-predictable behavior that cannot be described by analytical approaches. Models of spin glasses possess all of the previously mentioned characteristics: long relaxation times, a rough energy landscape, macroscopic degeneracy of ground states, and critical slowing down effect. All of these features together make it extremely difficult to study such systems.

### 2.2. Data

For the lattice N=L×L, there are $2^{2N}$ possible distributions of the exchange integral, starting with the distribution where all exchange interactions are antiferromagnetic $J_{k}=-1,\forall i$⇒$\sum_{k=1}^{2N}J_{k}=-2N$, passing through all possible combinations $\left\{J_{1},J_{2},J_{3},\ldots J_{2N}\right\}$ of exchange integrals and ending with the classical ferromagnetic model in which all interactions $J_{k}=1,\forall i$⇒$\sum_{k=1}^{2N}J_{k}=2N$. To find the dependence between the spatial distribution function of the exchange integral and the main average thermodynamic characteristics of the system, it is necessary to train the neural network for detecting different patterns of the mutual location of the exchange interaction values on the lattice and their influence on the macroscopic parameters of the system. For this purpose, the considered configuration of the spin glass with the given values $\left\{J_{1},J_{2},J_{3},\ldots J_{2N}\right\}$ and the temperature *T* should be fed to the input of the neural network. Then, it can be trained to predict the output mean energy 〈E〉 and magnetization 〈M〉 of the considered configuration of the spin glass. We would like to note that in the same way, it is possible to train a neural network to predict the probability density of states, residual entropy, heat capacity, susceptibility and other parameters characterizing the considered spin glass.

In order to train the neural networks, it was necessary to prepare datasets for training, validation and testing of neural network models. To this end, 60 temperatures from 0.1 to 6 in step 0.1 were calculated for each considered configuration of the spin glass. Simulations were performed using a parallel replica-exchange Monte Carlo (MC) method. There were 10,000 equilibration MC steps, then the energy and magnetization of the system were calculated and averaged over the next 100,000 MC steps according to Equations (2) and (3). To overcome the effects of critical slowing down and getting stuck in local minima, the system was simulated in parallel at 60 temperatures and the system configurations were exchanged every 1000 MC steps with a probability dependent on the system energy:

(4) $$\begin{array}{c} p\left(X\overset{}{\to }X^{\prime }\right)=\left\{\begin{array}{cc} 1 & \;\mathrm{if}\;\Delta \leq 0\\ \exp(\Delta ) & \;\mathrm{if}\;\Delta >0 \end{array}\right., \end{array}$$

where $\Delta =\left(1/T^{\prime }-1/T\right)\left(E^{\prime }-E\right)$, *E* and $E^{\prime }$ are the energies corresponding to *X* and $X^{\prime }$ configurations, respectively.

In total, two datasets each were calculated for two system sizes. The datasets contained 2N values of all interactions *J*, one value of temperature *T* and two output values of mean energy 〈E〉 and mean magnetization 〈M〉, therefore, a total of 2N+3 values. For the spin glass model with N=6×6 number of spins, the small dataset consisted of 834 configurations (with dimension of 50,040 × 75 since each spin glass configuration was calculated at 60 different temperature values) and the large one consisted of 41,405 configurations (2,484,300 × 75). For the model with N=10×10 number of spins, the small dataset consisted of 10,302 configurations (618,120 × 203) and the large of 43,596 configurations (2,615,760 × 203).

All configurations were randomly generated, with different constraints on the total sum of all interactions $\sum_{k=1}^{2N}J_{k}$. In the small datasets, the spin glass configurations were presented almost uniformly with respect to all possible values of the sum of all interactions $\sum_{k=1}^{2N}J_{k}$ from −2N to 2N in steps of 2. In large datasets, the distribution of spin glass configurations over the sum of all interactions tended to the corresponding values of binomial coefficients ∑k=12NJk=2N−2j∼j2N, where *j* is the number of negative interactions $J_{k}=-1$. All datasets were divided into train, validation and test subsets in proportions of 0.8:0.15:0.05.

### 2.3. Deep Neural Networks

**Figure 2:**
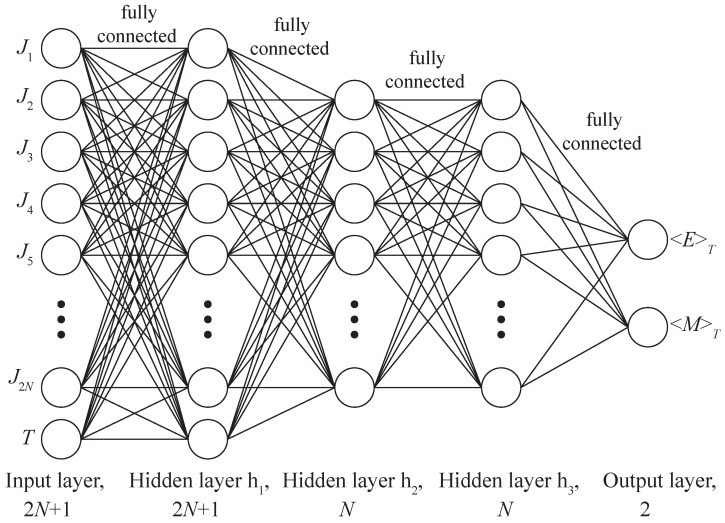
Deep Neural Network architecture FC4 (Fully Connected) with three hidden layers $h_{1}=2N+1$, $h_{2}=N$, $h_{3}=N$.

A deep neural network (DNN) is an artificial neural network (ANN) of forwarding propagation, i.e., multilayer perceptron, with more than one hidden layer. Similar to biological neurons with axons and dendrites, a DNN represents layers of artificial neurons with a given activation function, which are interconnected by trainable coefficients (see Figure 2). The first layer is called the input layer, the last one is the output layer, and all layers between them are called hidden layers. At the initial stage, the neural network is untrained, i.e., the linkage weights are set randomly and not optimized for the certain problem. The training of a neural network involves the adaptation of the network to the solution of a particular problem, carried out by adjusting the weight coefficients of each layer to minimize a given loss function L [31,32]. In this work, we use the mean squared error (MSE) as the loss function.

ANN training is performed using the error backpropagation method in two stages. During forward propagation, the input data are fed to the input of the ANN and then propagated through all the hidden layers to the output layer. The neurons of each layer receive data from the neurons of the previous layer, and their values are calculated using a matrix of weights *W*, bias *b* (5) and activation function *h* (6). The resulting values are transmitted to the next layer, i.e., the output of layer l−1 becomes the input of layer *l*, and so on, to the output layer of the network. A parametric rectified linear unit (PReLU) was used as the activation function *h*, which solved the problem of the so-called “dying ReLU”, when some neurons were simply turned off from training (7).

(5) $$\begin{array}{c} y^{\left[l\right]}=W^{\left[l\right]}h^{\left[l-1\right]}+b^{\left[l\right]}, \end{array}$$

(6) $$\begin{array}{c} h^{\left[l\right]}=f\left(y^{\left[l\right]}\right), \end{array}$$

(7) $$\begin{array}{c} f\left(y\right)=\left\{\begin{array}{cc} y & \;\mathrm{if}\;y>0\\ ay & \;\mathrm{if}\;y\leq 0 \end{array}\right., \end{array}$$

where *a* is a learnable parameter controlling the slope of the negative part of the function.

In the second step, all ANN weights are updated so as to minimize the loss function on a given dataset. For this purpose, the gradients for the variable parameters are calculated according to (8)–(10):

(8) $$\begin{array}{c} \frac{\partial L}{\partial y^{\left[l-1\right]}}={\left[W^{\left[l\right]}\right]}^{T}\frac{\partial L}{\partial y^{\left[l\right]}}f^{\prime }\left(y^{\left[l-1\right]}\right), \end{array}$$

(9) $$\begin{array}{c} \frac{\partial L}{\partial W^{\left[l\right]}}=\frac{\partial L}{\partial y^{\left[l\right]}}{\left[h^{\left[l-1\right]}\right]}^{T}, \end{array}$$

(10) $$\begin{array}{c} \frac{\partial L}{\partial b^{\left[l\right]}}=\frac{\partial L}{\partial y^{\left[l\right]}}. \end{array}$$

Then, the weights values and the bias are updated according to the calculated gradients:

(11) $$\begin{array}{c} W^{\left[l\right]}:=W^{\left[l\right]}-\alpha \frac{\partial L}{\partial W^{\left[l\right]}}, \end{array}$$

(12) $$\begin{array}{c} b^{\left[l\right]}:=b^{\left[l\right]}-\alpha \frac{\partial L}{\partial b^{\left[l\right]}}, \end{array}$$

where α is a learning rate parameter. This is the way the ANN is trained, during which with each step we descend to the global minimum of the convex loss function L with speed α.

## 3. Results and Discussion

Fully connected (FC) network architectures with different numbers and sizes of hidden layers were proposed as a baseline (see Figure 2). The following FC network architectures with one, two, three and four hidden layers were tested on datasets of the model with 6×6 size:

FC1: $h_{1}=73$;
FC2: $h_{1}=36$, $h_{2}=36$;
FC3: $h_{1}=73$, $h_{2}=36$;
FC4: $h_{1}=73$, $h_{2}=36$, $h_{3}=36$;
FC5: $h_{1}=73$, $h_{2}=36$, $h_{3}=36$, $h_{4}=36$.

The effect of the number and size of DNN’s hidden layers *h* on the quality and speed of learning was investigated. Five different fully connected neural network architectures (FC1–FC5) were trained on small and large datasets. The average learning time per epoch in seconds was calculated for them, and the root mean squared errors (RMSE) of the mean energy and magnetization was calculated with the DNN from the initial values obtained with the replica-exchange Monte Carlo (see Table 1). For the big dataset, the number of epochs was 500, and for the small dataset it was 1000.

The architecture FC4 with three hidden layers, shown in Figure 2, showed optimal results, yielding only slightly in accuracy on a small dataset of a network with four hidden layers (FC5). Further results will be compared with these two architectures.

**Table 1:** Comparison of average single epoch learning rate and RMSE of mean energy 〈E〉 and magnetization 〈M〉 for small and big datasets of FC1–FC5 fully connected neural networks.

	FC1	FC2	FC3	FC4	FC5
RMSE${}_{small}\langle E\rangle$	2.483	2.378	2.370	2.323	2.321
RMSE${}_{small}\langle M\rangle$	0.133	0.103	0.101	0.088	0.088
Time${}_{small}$, c	3	3	3	3	4
RMSE${}_{big}\langle E\rangle$	8.998	2.655	2.340	1.938	1.938
RMSE${}_{big}\langle M\rangle$	0.271	0.179	0.093	0.065	0.065
Time${}_{big}$, c	26	28	31	35	45

To improve the speed and accuracy of the calculations, we investigated DNNs whose architectures would transmit information about the spatial arrangement of the connections on the square lattice. We proposed to replace fully connected hidden layers with layers in which neurons would be connected similarly to spins on a square lattice. The speed improvement is achieved by having fewer training weights in the layers compared to fully connected architectures.

**Figure 3:**
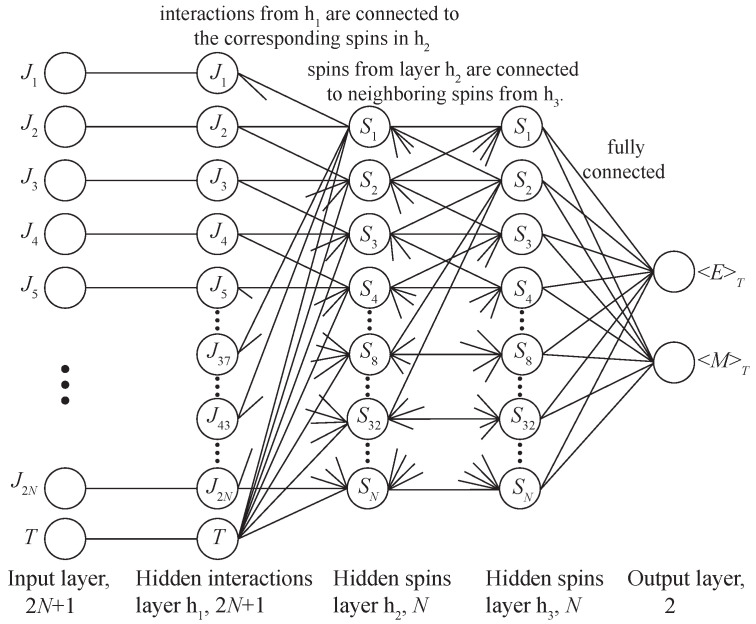
The proposed architecture of the CC2 deep neural network with three hidden layers $h_{1}=2N+1$, $h_{2}=N$, $h_{3}=N$. The first hidden layer $h_{1}$ connected with the layer $h_{2}$ in the same way as bonds $J_{k}$ connect spins $S_{j}$ on a square lattice (neurons of the layer $h_{1}$ have 2 outputs, and $h_{2}$ have 5 inputs). The second hidden layer $h_{2}$ is connected with the third $h_{3}$ just like the neighboring spins are connected with each other on the square lattice (neurons of the layer $h_{2}$ have 5 outputs, and $h_{3}$ 5 inputs, see Figure 1).

Two architectures of DNN with two levels of spin lattice abstraction were considered CC1 and CC2 (CC—Custom Connected). The first architecture CC1 proposes to consider the first hidden layer $h_{1}$ as virtual bonds, and the second layer $h_{2}$ as virtual spins. In such a network, all neurons of layer $h_{1}$, except the temperature neuron, are connected to the corresponding neurons of layer $h_{2}$ in the same way that bonds in a square lattice are connected to spins. For example, spin $S_{1}$ has four bonds: $J_{1},J_{2},J_{L\times L+1=37},J_{L\times (L+1)+1=43}$ (Figure 1), so in the neural network, neurons 1,2,37 and 43 of layer $h_{1}$ will be connected to the first neuron of layer $h_{2}$, see Figure 3. Thus, each neuron of the $h_{1}$ layer is connected to two corresponding neurons of the $h_{2}$ layer, except for the temperature neuron of the $h_{1}$ layer, which is connected to all neurons of the $h_{2}$ layer. In such an architecture, the connection between layers $h_{2}$ and $h_{3}$, as well as $h_{3}$ and the output layer, was fully connected.

The second architecture of CC2 has the same approach as in CC1, except for the connections between layers $h_{2}$ and $h_{3}$. In CC2, it was proposed to consider the $h_{3}$ layer also as virtual spins, and to connect the $h_{2}$ layer with $h_{3}$ in a manner similar to the neighboring spins in a square lattice (Figure 1). For example, if spin $S_{2}$ is a neighbor of spins $S_{1},S_{3},S_{L+2=8},S_{(L\times L-1)+2=32}$, it means that the second neuron of layer $h_{3}$ will be connected with neurons 1,3,8,32 and also with neuron 2 (See Figure 3). Hidden layer $h_{3}$ is fully connected with the output layer. The connection between layers $h_{3}$ and the output layer was fully connected.

**Figure 4:**
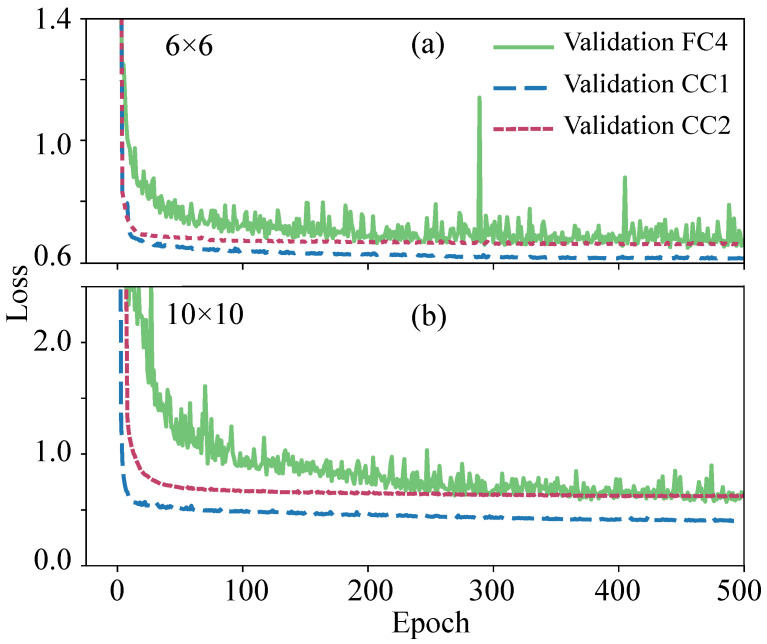
Dependence of the loss function value L during validation on epoch number for the FC4 and the proposed CC1 and CC2 architectures, trained on a large dataset for the model N=6×6 (**a**); N=10×10 (**b**).

To study the accuracy of the proposed DNN architectures with a baseline solution, the neural networks were trained and tested on big datasets for spin glass systems N=6×6 and N=10×10. To control the overfitting of the neural networks during training, the loss function L was calculated on a validation sample that was not involved in the training. The graph of the loss function value L as a function of the number of training epochs is shown in Figure 4. This figure shows that for 500 epochs, overfitting does not occur for any of the architectures considered. However, there is a large difference in the speed and accuracy of learning.

**Figure 5:**
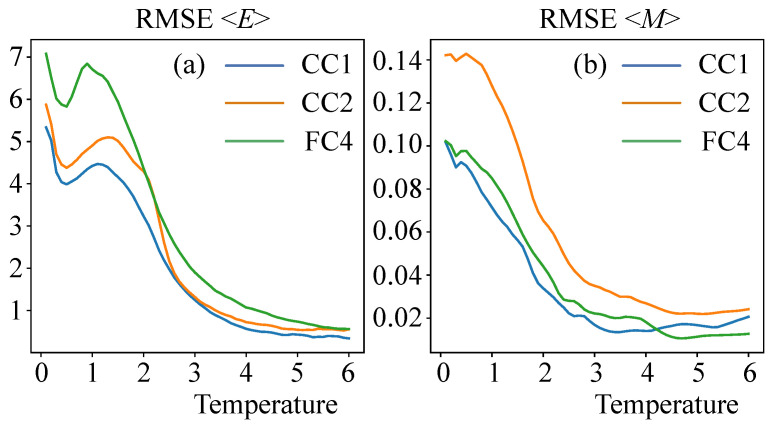
Dependence of the root mean squared error (RMSE) on the temperature for the average energy 〈E〉 (**a**) and magnetization 〈M〉 (**b**) for neural networks of different architectures (FC4, CC1 and CC2).

The results of the work of neural networks were scored by root mean squared error (RMSE) of the average energy 〈E〉 and magnetization 〈M〉. The resulting RMSE values depending on DNN architecture and system size are presented in Table 2. The table shows that the CC1 architecture was the most accurate, reducing the average energy calculation error by one-and-a-half times compared to the fully connected architectures. Figure 5 also shows the dependence of the root mean squared error (RMSE) on temperature for the average energy 〈E〉 and magnetization 〈M〉 for neural networks of different architectures. It is well noticeable that the computational error increases with decreasing temperature. This is due to the complexity of calculating the ground states of the spin glass models. To reduce the error, it is possible to use, for example, the approach described in [26], which allows using a restricted Boltzmann machine to calculate the ground states of the spin glass systems. It is also clear that the CC1 architecture reduces the error, this difference can be seen especially at small temperatures.

**Table 2:** Comparison of the results of the root mean squared error (RMSE), calculated by different architectures of DNN, of the mean energy 〈E〉 and magnetization 〈M〉 for spin glasses of sizes 6×6 and 10×10.

Architectures	RMSE 〈E〉		RMSE 〈M〉	
	6×6	10×10	6×6	10×10
FC4	1.9991	3.7660	0.0601	0.0491
FC5	2.0045	3.8168	0.0688	0.0492
CC1	1.4854	2.6071	0.0642	0.0443
CC2	1.7674	3.0173	0.0673	0.0581

**Figure 6:**
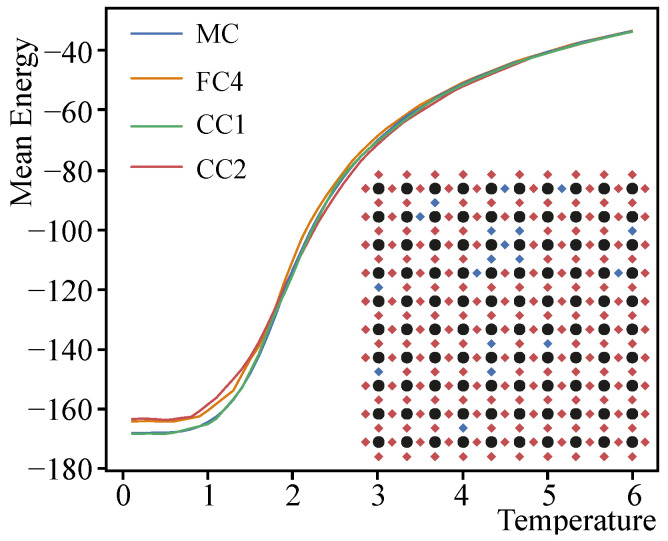
Example of average energy calculation using replica-exchange MC as well as neural networks of different architectures (FC4, CC1 and CC2). The configuration of the calculated spin glass is shown in the lower right corner of the figure. Black circles denote spins, red rhombuses denote J=1 bonds, and blue J=−1 bonds.

Figure 6 shows an example of mean energy calculation using a replica-exchange MC and DNNs of different architectures (FC4, CC1 and CC2). The configuration of the calculated spin glass is shown in the corner of the figure. It can be seen that the calculation result of the network with the CC1 architecture is almost identical to that obtained with the replica-exchange MC, while the results of the networks with the FC4 and CC2 architectures have some deviations at low and medium temperatures.

## 4. Conclusions

In this paper, we presented a method for calculating the values of thermodynamic averages of a frustrated spin glass model using deep neural networks. The influence of the neural network architecture on the speed and accuracy of calculations for spin glass models N=6×6 and 10×10 with different distributions of the exchange integral *J* was studied. Specific neural network architectures have been proposed to increase the accuracy and reduce the error compared to fully connected models. The use of trained neural networks can significantly reduce the time compared to classical numerical approaches when modeling spin glass systems. This approach, after appropriate training, allows modeling of any global characteristics of spin glass including probability density of states, residual entropy, heat capacity, susceptibility, and so on.

With the help of deep neural networks, it has become possible to calculate the global characteristics of the system quite accurately based on the microarchitecture (a certain distribution of edges values) of a particular configuration of the spin glass. Based on the obtained results, we can conclude that neural network architectures that simulate the structure of spin lattices are better adapted to the calculation of spin glass models. In further development of this topic of using neural networks in the modeling of complex magnetic systems, it is interesting to consider convolutional models of neural networks on lattices. As convolution should be performed not by nodes, but by bonds, the recently proposed graph neural networks (GCN) [33], which allow to move away from clearly fixed lattice sizes and calculate systems of any size, are interesting. This will make it possible to train such networks on small lattices computed by various exact methods [34], and to extend the obtained functional regularities to large systems.

## Data Availability

The data of the current study are available from the corresponding author.
